# Safety and Pharmacokinetics of CD101 IV, a Novel Echinocandin, in Healthy Adults

**DOI:** 10.1128/AAC.01627-16

**Published:** 2017-01-24

**Authors:** Taylor Sandison, Voon Ong, Jonathan Lee, Dirk Thye

**Affiliations:** Cidara Therapeutics, Inc., San Diego, California, USA

**Keywords:** CD101, Candida, antifungal agents, clinical trials, echinocandin, pharmacokinetics

## Abstract

CD101 IV is a novel echinocandin with distinctive pharmacokinetic properties that is being developed as a once-weekly treatment for candidemia and invasive candidiasis. CD101 has potent *in vitro* activity and *in vivo* efficacy against a broad range of Candida and Aspergillus species. The primary objective of two randomized, double-blind, placebo-controlled, dose-escalation studies in healthy adults was to determine the safety and tolerability of CD101 IV. Sequential cohorts of 8 subjects (*n* = 6, active; *n* = 2, placebo) were administered single (50, 100, 200, 400 mg) or multiple once-weekly (100 mg given once weekly for two weeks [×2], 200 mg ×2, 400 mg ×3) doses of CD101 IV infused over 1 h. There were no deaths, serious adverse events (SAEs), severe adverse events (AEs), or withdrawals from the study due to an AE. The majority of AEs were mild, and all completely resolved. There was a higher incidence of total AEs and mild transient infusion reactions in the 400-mg ×3 dose group. There were no clinically meaningful trends in postbaseline laboratory abnormalities and no safety issues related to electrocardiograms, vital signs, or physical exams. CD101 showed dose-proportional plasma exposures, minor accumulation (30% to 55%), low apparent clearance (<0.28 liter/h), long half-life (*t*_1/2_) (>80 h), and minimal urine excretion. CD101 IV was safe and well tolerated at single and multiple doses of up to 400 mg given once weekly for 3 weeks and exhibited a long *t*_1/2_, minimal accumulation over several weeks, negligible renal excretion, and high plasma exposures enabling once-weekly dosing.

## INTRODUCTION

CD101 IV, a new echinocandin antifungal drug, is being developed for candidemia and invasive candidiasis. These life-threatening infections represent a significant public health issue, with a mortality rate of >40%, particularly in highly vulnerable patient populations (1–4). Because of increasing resistance to existing antifungal drugs, there is an urgent need to develop new and more-effective antifungal agents (5–9).

More than 50% of all Candida infections are caused by non-albicans Candida species, including Candida glabrata, which is the most common non-albicans Candida species in North America. The incidence of infections caused by C. glabrata has increased by >4-fold over the past 20 years (10) at some sites in the United States, and the frequency of C. glabrata as a cause of bloodstream infections has increased from 18% (2008/2009) to 25% (2010/2011) (11). Azole-resistant C. glabrata is recognized as a serious public health threat (9, 12, 13) in North America, and its incidence has increased from 9% (1992 to 2001) to 14% (2001 to 2007) (11, 14–18) and is as high as 30% (5). Because of its haploid genome, C. glabrata has the potential to rapidly develop resistance to antifungal agents in multiple drug classes, including echinocandins (7, 15, 19). The emergence of multidrug-resistant C. glabrata is considered a major public health issue because neither azoles nor currently available echinocandins would be appropriate treatments, and the only alternative (high-dose amphotericin B) is associated with renal toxicity, particularly in older, critically ill patients with sepsis (20–24).

CD101 has potent *in vitro* activity against a broad spectrum of clinically important Candida spp. (25) and is active in animal models of Candida infection (26), including azole-resistant Candida isolates. Like other echinocandins, CD101 displays a concentration-dependent pattern of fungicidal activity *in vitro* and *in vivo* and a favorable safety profile *in vivo* (26). These characteristics, combined with the pharmacokinetic (PK) properties of CD101, allow high front-loaded drug exposures and make CD101 a potentially better option than other echinocandins for the treatment of infections by multidrug-resistant C. glabrata strains (27, 28).

Pharmacokinetic evaluations of CD101 in multiple animal species have been conducted (V. Ong, K. D. James, S. Smith, B. R. Krishnan, submitted for publication). These are the first studies to examine the safety profile of CD101 in humans, assessing 4 dose regimens for up to 3 weeks.

## RESULTS

### Patient disposition and analysis populations.

The phase 1 single-ascending-dose study enrolled 32 subjects, and the multiple-ascending-dose study enrolled 24 subjects. One subject (100-mg group) withdrew from the single-ascending-dose study on day 3 due to a family emergency. All subjects in both studies were included in the safety and PK analysis populations.

### Patient demographics and baseline medical characteristics.

In the single- and multiple-ascending-dose studies, subjects were predominately white (97% and 88%) and Hispanic or Latino (94% and 75%) and had values for mean body mass index of 28.1 and 27.2 kg/m^2^, mean age of 43.2 and 42.8 years, and median age of 43.0 and 46.0 years, respectively (Table 1). Overall, males and females were equally represented in both studies.

**TABLE 1:** Demographic and baseline characteristics (safety population)

Characteristic	Value(s)	
	Single-ascending-dose study (*n* = 32)	Multiple-ascending-dose study (*n* = 24)
Age, yrs		
Mean (SD)	43.2 (7.9)	42.8 (9.4)
Median (range)	43.0 (25–54)	46.0 (22–54)
Sex, no. (%) of subjects		
Male	17 (53)	12 (50)
Female	15 (47)	12 (50)
Ethnicity, no. (%) of subjects		
Hispanic or Latino	30 (94)	18 (75)
Non-Hispanic or Latino	2 (6)	6 (25)
Race, no. (%) of subjects		
White	31 (97)	21 (88)
Black or African American	0	3 (13)
American Indian or Alaska Native	1 (3)	0
Body mass index, kg/m^2^		
Mean (SD)	28.1 (2.6)	27.2 (2.9)
Median (range)	28.3 (22.7–31.9)	27.1 (22.3–31.6)

The demographic and baseline characteristics of the subjects in the CD101 dose groups and the pooled placebo groups were similar.

### Safety outcomes.

There were no serious adverse events (SAEs), severe adverse events (AEs), withdrawals due to an AE, or deaths.

In the single-ascending-dose study, there were no dose-response trends observed for treatment-emergent adverse events (TEAEs) or TEAEs related to study drug across the 4 CD101 dose cohorts. TEAEs occurred in 3 (50%) subjects in cohort 1, 0 (0%) subjects in cohort 2, 3 (50%) subjects in cohort 3, 1 (17%) subject in cohort 4, and 5 (63%) subjects in the placebo group. No individual TEAEs occurred in >1 subject within each CD101 dose cohort. The most common system organ classes with respect to symptoms were gastrointestinal disorders (2 [33%] subjects in cohorts 1 and 3 and 2 [25%] subjects in the placebo group), general disorders and administration site conditions (2 [33%] subjects in cohort 3 and 4 [50%] subjects in the placebo group), and musculoskeletal and connective tissue disorders (2 [33%] of the CD101 subjects in cohort 1, 2 subjects in cohort 3, 1 subject in cohort 4, and 2 subjects in the placebo group). Moderate TEAEs occurred in 1 subject (ear pain) in cohort 3, 1 subject (headache) in cohort 4, and 3 subjects (abdominal pain—lower, vessel puncture site swelling, viral infection, arthralgia, and dysuria) in the placebo group.

In the multiple-ascending-dose study, there was a higher incidence of TEAEs and a higher total number of TEAEs in cohort 3 (400 mg given once weekly for 3 weeks [×3 weeks]; 4/6, 67%) than in cohort 1 (100 mg ×2 weeks; 3/6, 50%) and cohort 2 (200 mg ×2 weeks; 2/6, 33%) and the placebo group (2/6, 33%). The most common system organ classes with respect to symptoms were gastrointestinal disorders (1 [17%] subject in cohort 1, 4 [67%] subjects in cohort 3, and 1 [17%] subject in the placebo group) and vascular disorders (1 [17%] subject in cohort 1 and 3 [50%] subjects in cohort 3). Four subjects in the CD101 cohorts experienced mild, transient infusion reactions, characterized by flushing, feeling hot, nausea, and chest tightness (Table 2). These infusion reactions were associated primarily with cohort 3 and were most common with the third dose. In general, these reactions occurred and disappeared within minutes of infusion without interruption or discontinuation of study drug. No infusion reactions were observed with dose 1 in any cohort. One subject in cohort 3 had an infusion reaction with dose 2 and dose 3. No intervention was required for the symptoms, and there were no sequelae. The only other individual TEAEs in ≥2 subjects across the CD101 dose cohorts were mild or moderate constipation (2 subjects) and mild or moderate myalgia (2 subjects), both in cohort 3 (Table 2). TEAEs related to study drug occurred in 1 subject in cohorts 1 and 2, 4 subjects in cohort 3, and 1 subject in the placebo group; the only non-infusion reaction TEAE related to study drug that occurred in ≥2 subjects was mild or moderate constipation (2 subjects). Moderate TEAEs occurred in 1 subject (arthropod bite and pruritus) in cohort 2, 2 subjects (constipation and myalgia) in cohort 3, and 1 subject (pruritus) in the placebo group.

**TABLE 2:** Subjects with treatment-emergent adverse events by system organ class and preferred term (safety population)^*a*^

Preferred term	No. (%) of subjects (*n* = 6)								
	Single-ascending-dose study					Multiple-ascending-dose study			
	CD101 dose (mg)				Pooled placebo (*n* = 8)	CD101 dose (mg)			Pooled placebo
	50	100	200	400		100	200	400	
All TEAEs	3 (50)	0	3 (50)	1 (17)	5 (63)	3 (50)	2 (33)	4 (67)	2 (33)
Abdominal distension	0	0	0	0	0	0	0	1 (17)	0
Abdominal pain	0	0	1 (17)	0	0	0	0	1 (17)	0
Abdominal pain lower	0	0	0	0	1 (13)	0	0	0	0
Alopecia	0	0	0	0	0	0	0	1 (17)	0
Aphthous stomatitis	0	0	1 (17)	0	0	0	0	1 (17)	0
Arthralgia	0	0	0	0	2 (25)	0	0	0	0
Arthropod bite	0	0	0	0	0	0	1 (17)	0	0
Back pain	0	0	1 (17)	0	0	0	0	1 (17)	0
Blood pressure increased	0	0	0	0	0	1 (17)	0	0	0
Catheter site pruritus	0	0	1 (17)	0	0	0	0	0	0
Chest discomfort	0	0	0	0	0	0	0	2 (33)	0
Chills	0	0	1 (17)	0	0	0	0	0	0
Constipation	1 (17)	0	0	0	0	0	1 (17)	2 (33)	0
Diarrhea	1 (17)	0	0	0	0	0	0	1 (17)	0
Dizziness	0	0	1 (17)	0	1 (13)	0	0	1 (17)	0
Dyspnea	0	0	0	0	0	0	0	1 (17)	0
Dysuria	0	0	0	0	1 (13)	0	0	0	0
Ear pain	0	0	1 (17)	0	0	0	0	0	0
Feces hard	0	0	1 (17)	0	0	0	0	0	0
Feeling hot	0	0	0	0	2 (25)	1 (17)	0	1 (17)	0
Flushing	0	0	0	0	0	1 (17)	0	3 (50)	0
Glossodynia	0	0	0	0	0	0	0	0	1 (17)
Hematochezia	0	0	0	0	0	0	0	1 (17)	0
Headache	0	0	1 (17)	1 (17)	2 (25)	0	0	0	0
Hyperhidrosis	0	0	0	0	0	0	0	1 (17)	0
Hypoesthesia	0	0	0	0	0	0	0	1 (17)	1 (17)
Infusion site pain	1 (17)	0	0	0	0	0	0	0	0
Insomnia	0	0	1 (17)	0	0	0	0	0	0
Lymphadenopathy	0	0	0	0	1 (13)	0	0	0	0
Micturition urgency	0	0	0	0	1 (13)	0	0	0	0
Musculoskeletal chest pain	0	0	0	0	0	0	0	1 (17)	0
Myalgia	0	0	1 (17)	0	0	0	0	2 (33)	0
Nausea	0	0	1 (17)	0	0	0	0	0	0
Nodule	0	0	0	0	0	0	0	0	1 (17)
Pain in jaw	0	0	1 (17)	0	0	0	0	0	0
Papule	0	0	0	0	0	0	0	0	1 (17)
Paresthesia	0	0	0	0	1 (13)	0	0	0	0
Pollakiuria	0	0	1 (17)	0	1 (13)	0	0	0	0
Presyncope	0	0	0	0	0	0	0	1 (17)	0
Proctalgia	0	0	0	0	0	0	0	1 (17)	0
Pruritus	0	0	0	0	0	0	1 (17)	0	1 (17)
Rash (papular)	0	0	0	0	0	1 (17)	0	0	1 (17)
Rhinorrhea	0	0	0	0	0	0	1 (17)	0	0
Tachycardia	0	0	1 (17)	0	0	0	0	0	0
Throat tightness	0	0	0	0	0	0	0	1 (17)	0
Tongue eruption	0	0	0	0	0	0	0	0	1 (17)
Toothache	0	0	0	0	1 (13)	0	0	0	0
Upper respiratory tract infection	0	0	0	0	0	0	0	1 (17)	0
Urinary tract infection	0	0	0	0	0	0	0	1 (17)	0
Urine output increased	0	0	0	0	1 (13)	0	0	0	0
Vessel puncture site bruise	0	0	0	0	2 (25)	0	0	0	0
Vessel puncture site pain	0	0	0	0	2 (25)	0	0	0	1 (17)
Vessel puncture site swelling	0	0	0	0	1 (13)	0	0	0	0
Viral infection	0	0	0	0	1 (13)	0	0	0	0
Vision blurred	0	0	0	0	0	1 (17)	0	0	0

a TEAEs, treatment-emergent adverse events. Adverse events were linked to preferred terms from the *Medical Dictionary for Regulatory Activities*, version 18.0.

There were no clinically meaningful trends in postbaseline laboratory abnormalities (Table 3), clinically meaningful shifts from normal at baseline to outside normal range postbaseline, or AEs for any laboratory parameter and no clinically significant abnormal vital signs or electrocardiogram (ECG) results.

**TABLE 3:** Laboratory findings

Laboratory result	No. (%) of subjects (*n* = 6)								
	Single-ascending-dose study^*a*^					Multiple-ascending-dose study^*b*^			
	CD101 dose (mg)				Pooled Placebo (*n* = 8)	CD101 dose (mg)			Pooled Placebo
	50	100	200	400		100	200	400	
Hematology^*c*^									
Normal	78 (87)	80 (99)	89 (99)	88 (98)	115 (96)	136 (94)	137 (95)	198 (100)	159 (98)
Abnormal—not clinically significant	12 (13)	2 (2)	1 (1)	2 (2)	5 (4)	8 (6)	7 (5)	0	3 (2)
Abnormal—clinically significant	0	0	0	0	0	0	0	0	0
Chemistry^*c*^									
Normal	461 (96)	432 (100)	465 (97)	475 (99)	619 (96.5)	699 (97)	696 (97)	969 (98)	790 (98)
Abnormal—not clinically significant	19 (4)	0	16 (3)	5 (1)	18 (3)	21 (3)	24 (3)	21 (2)	20 (3)
Abnormal—clinically significant	0	0	0	0	2 (0.5)	1^*d*^	0	0	0

a A total of 5 blood draws were performed for each subject on days 2, 4, 7, 14, and 21.

b A total of 8 blood draws were performed for each subject in the 100-mg and 200-mg CD101 dose cohorts and placebo group on days 2, 4, 7, 9, 11, 14, 21, and 28; a total of 11 blood draws were performed for each subject in the 400-mg CD101 dose cohort on days 2, 4, 7, 9, 11, 14, 16, 18, 21, 28, and 35.

c Data represent 3 parameters for hematology (hemoglobin, white blood cell count, and platelets) and 15 parameters for chemistry (calcium, chloride, bicarbonate, potassium, albumin, blood urea nitrogen, creatinine, glucose, alkaline phosphatase, aspartate aminotransferase, alanine aminotransferase, total bilirubin, direct bilirubin, protein, and sodium).

d This result represents a low glucose level on day 2—the subject had no clinical symptoms of hypoglycemia. The value was determined to be abnormal-clinically significant *a priori* at the start of the study; however, during the study, the lead clinician did not consider the abnormal laboratory finding to be clinically significant.

### Pharmacokinetic evaluation.

All CD101-treated and placebo-treated subjects in the single-ascending-dose and multiple-ascending-dose studies provided blood (plasma) and urine samples for PK analysis. All samples from CD101-treated subjects were analyzed, with the exception of the one subject from the 100-mg group who withdrew from the single-ascending-dose study. Tables 4 and 5 give the arithmetic means and SD for the calculated PK parameters for the single- and multiple-ascending-dose studies, respectively.

**TABLE 4:** Arithmetic means and standard deviations of pharmacokinetic parameters from the single-ascending-dose study^*a*^

Parameter	Value for indicated dose (mg)							
	50		100		200		400	
	Mean	SD	Mean	SD	Mean	SD	Mean	SD
*C*_max_ (μg/ml)	2.76	0.574	4.86	0.561	10.9	2.17	22.7	3.59
AUC_0–168_ (μg · h/ml)	145	20.4	254	22.9	592	66.8	1160	170
*t*_1/2_ (h)	133	7.62	146	3.82	125	13.0	129	18.6
Cl (liters/h)	0.225	0.0378	0.240	0.0196	0.219	0.0230	0.226	0.0421
*V*_ss_ (liters)	36.0	5.68	42.9	4.07	34.4	4.99	35.9	5.59
*V_z_* (liters)	43.2	8.20	50.6	4.96	39.7	6.99	41.6	7.59

a SD, standard deviation; *V*_ss_, volume of distribution at steady state; *V_z_*, apparent volume of distribution during terminal phase.

**TABLE 5:** Arithmetic means and standard deviations of pharmacokinetic parameters from the multiple-ascending-dose study

Dose	Value for indicated dose (mg)^*a*^											
	100				200				400			
	Day 1		Day 8		Day 1		Day 8		Day 1		Day 15	
	Mean	SD	Mean	SD	Mean	SD	Mean	SD	Mean	SD	Mean	SD
*C*_max_ (μg/ml)	5.67	0.887	6.49	0.654	10.6	1.93	12.4	3.45	22.7	4.87	30.5	13.1
AUC_0–168_ (μg · h/ml)	299	27.4	390	44.1	570	125	813	225	1190	229	1840	323
*t*_1/2_ (h)	79.1	4.04	158	15.5	81.3	4.30	140	13.2	81.0	7.92	152	29.5
Cl (liters/h)	0.258	0.0218	0.149	0.0216	0.279	0.0734	0.155	0.0441	0.268	0.0573	0.126	0.0179
*V*_ss_ (liters)	28.8	3.59	29.3	3.70	31.7	7.72	28.5	7.18	29.6	6.35	25.4	7.08
*V_z_* (liters)	29.5	3.23	33.8	4.34	32.8	9.18	30.9	7.35	31.2	6.33	27.9	8.78

a SD, standard deviation.

**FIG 1:**
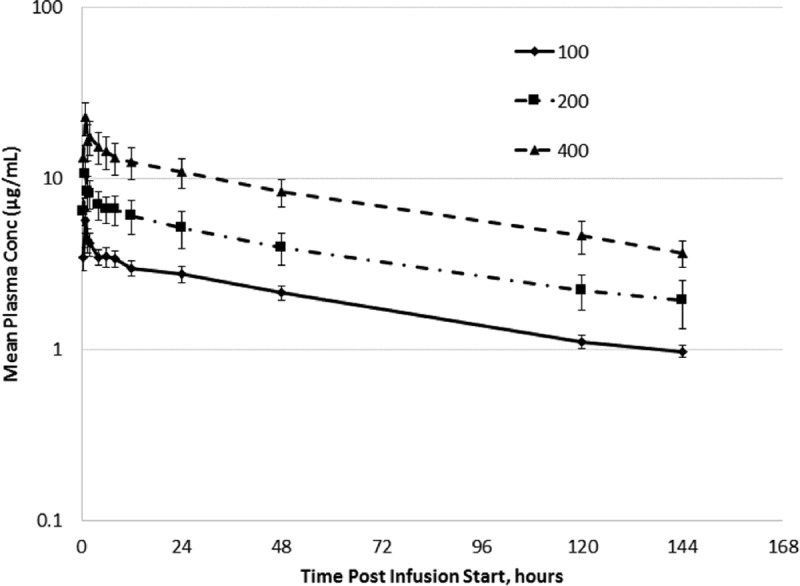
Arithmetic mean ± SD plasma CD101 concentration-time profiles following administration of CD101 IV infusion (1 dose of 100 mg, 200 mg, or 400 mg).

Pharmacokinetic results in the single-ascending-dose study were consistent with the PK results following the first dose of study drug in the multiple-ascending-dose study for each dose cohort (Fig. 1). Across the doses studied in the single-ascending-dose study, mean maximum plasma concentration (*C*_max_) values ranged from 2.76 to 22.7 μg/ml and the corresponding values for mean area under the concentration-time curve from time zero to 168 h (AUC_0–168_) ranged from 145 to 1,160 μg · h/ml. Both the *C*_max_ and AUC values increased in a dose-proportional manner, and mean half-life (*t*_1/2_) values of >80 h were observed through the first week (up to day 7) of plasma sampling (a longer terminal *t*_1/2_ of 125 to 146 h was calculated by incorporating data from later [days 14 and 21] sampling times).

In the multiple-ascending-dose study, exposures following the first dose were comparable to that observed in the single-ascending-dose study, with AUC and *C*_max_ values increasing in a dose-proportional manner. Values for terminal elimination *t*_1/2_, total body clearance (Cl), volume of distribution, and volume of distribution at steady state were similar across the CD101 dose levels. Similarly to day 1, values for the peak and extent of exposure to CD101 on day 8 increased with increasing doses in a ratio of 1:2.

**FIG 2:**
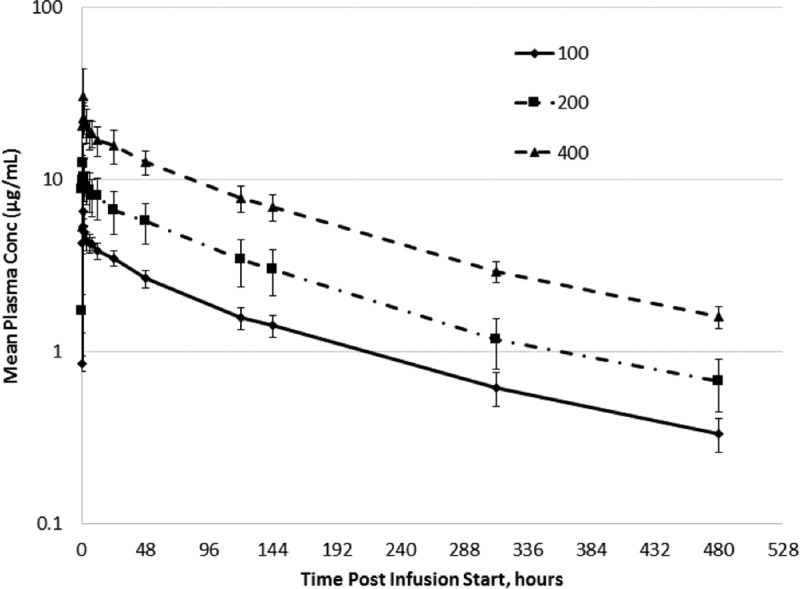
Arithmetic mean ± SD plasma CD101 concentration-time profiles following weekly administration of CD101 IV infusion (second dose of 100 mg, second dose of 200 mg, or third dose of 400 mg).

Plasma CD101 concentrations in the multiple-ascending-dose study were detectable in all subjects through 144 h postdose on day 1, 480 h postdose on day 8 (cohort 1 and cohort 2), and 480 h postdose on day 15 (cohort 3). Figure 2 shows the mean concentration-time profile following the second (100- or 200-mg) or third (400-mg) dose of CD101 in the multiple-ascending-dose study for each dose cohort. CD101 plasma concentrations were higher on day 8 (cohort 1 and cohort 2) and day 15 (cohort 3) than on day 1. CD101 trough concentrations on day 7 (144 h postdose), following the first and third (last) dose, were approximately 4 and 7 μg/ml, respectively. The highest mean plasma concentrations were observed 1 h after the start of infusion. Peak exposure was marginally (14% to 34%) higher following CD101 IV infusion on day 8 (cohort 1 and cohort 2) and day 15 (cohort 3) than on day 1.

The extent of exposure (AUC_0–168_) was higher on day 8 than on day 1 in cohort 1 (390 versus 299 μg · h/ml, respectively) and cohort 2 (813 versus 570 μg · h/ml, respectively) and higher on day 15 (1,840 μg · h/ml) than on day 1 (1,190 μg · h/ml) in cohort 3.

The mean volume of distribution (range, 28 to 34 liters) and the mean volume of distribution at steady state (range, 25 to 32 liters) were similar following administration of CD101 IV at all 3 dose levels on days 1, 8, and 15. Mean Cl across the 3 CD101 IV infusions on day 1 (range, 0.258 to 0.279 liter/h) was double that of days 8 and 15 (range, 0.126 to 0.155 liter/h). Mean terminal elimination *t*_1/2_ following CD101 IV infusion on days 8 and 15 (range, 140 to 158 h) was double that of day 1 (range, 79 to 81 h). The lower Cl and higher elimination *t*_1/2_ values reflect the longer PK sampling interval (480 h) following administration of the last dose (day 8 or 15) than following administration of the first dose (144 h).

A small amount (≤0.26%) of CD101 was excreted in urine. However, the cumulative amount of CD101 excreted in urine was approximately 3 times higher on days 8 and 15 than on day 1 in cohort 1 (151.4 versus 52.0 μg), cohort 2 (400.7 versus 145.0 μg), and cohort 3 (987.5 versus 330.6 μg), likely due to the contribution from the first or second dose. The mean renal clearance of CD101 ranged from 0.1877 ml/h in cohort 1 on day 1 to 0.5965 ml/h in cohort 3 on day 15, indicating that renal clearance does not play a significant role in Cl of CD101.

## DISCUSSION

These randomized, double-blind, placebo-controlled, ascending-dose studies evaluated the safety and PK of CD101 for a single dose of up to 400 mg and for multiple doses of up to 400 mg given once weekly for 3 weeks in healthy adults. CD101 was safe and well tolerated at all dose levels tested.

There were no SAEs, severe AEs, or withdrawals from study due to AEs, and most TEAEs were mild in severity. In the single-ascending-dose study, there was no dose response observed for TEAEs or TEAEs related to study drug across the 4 dose cohorts (50, 100, 200, and 400 mg). In the multiple-ascending-dose study, there was a tendency toward higher rates of AEs in cohort 3, the group administered the highest dose of CD101 for the longest duration (400 mg once weekly for 3 weeks). The most common AEs in cohort 3 were moderate constipation (2 subjects), mild nausea (2 subjects), mild chest discomfort (2 subjects), mild or moderate myalgia (2 subjects), and mild flushing (3 subjects). No AEs for any hematology, chemistry, or urinalysis (UA) parameters were reported in cohort 3. Mild infusion reactions, characterized by flushing, nausea, and chest discomfort, were most common with the third dose of 400 mg of CD101, occurring and disappearing within minutes of infusion initiation, without interruption or discontinuation of the study drug infusion. It is possible that the few infusion reactions observed were part of a previously identified echinocandin class effect but perhaps with reduced severity. Infusion reactions characterized by flushing are expected AEs for anidulafungin and are the reason that the anidulafungin infusion rate limit is 1.1 mg/min (29). The CD101 infusion rate at the 400-mg dose is over 6 mg/min.

Overall, no trends or clinical safety concerns with respect to abnormal hematology, chemistry, or UA values were observed in either treatment group and there was no dose response for laboratory values observed across CD101 dose groups in either study.

Since none of the dose regimens tested in these two studies was limited by toxicity, target attainment analyses were used to determine appropriate dosing for the ongoing phase 2 study in patients with candidemia (30). The two dose regimens chosen for this study are 400 mg once weekly and 400 mg once followed by 200 mg once weekly.

The plasma PK of CD101 was generally well characterized in the single-ascending-dose and multiple-ascending-dose studies. In both studies, AUC and *C*_max_ increased in a dose-proportional manner and levels of total Cl were low and comparable throughout the dose levels. The fraction of dose excreted was <1% at all dose levels, indicating the minor contribution of renal clearance in CD101 excretion. Pharmacokinetic results in the single-ascending-dose and multiple-ascending-dose studies were consistent with projected human PK results from nonclinical studies (26).

CD101 has a longer *t*_1/2_ (approximately 80 h following the first dose and approximately 150 h following the second or third dose) than any currently available echinocandin, enabling CD101 IV to be dosed less frequently. Two weekly doses of CD101 IV may provide a full course of echinocandin treatment for the majority of patients with candidemia, thus precluding the need to step down to an azole to complete treatment. This is particularly important in the context of increasing rates of infection caused by azole-resistant C. glabrata, which can be difficult to identify at many United States hospitals.

Antifungal agents, e.g., echinocandins, that demonstrate concentration-dependent killing (29–32) and also exhibit low Cl and a favorable safety profile can be administered much like a front-loaded regimen, with the advantage that effective drug exposures are reached immediately after dosing and then decrease over time. From a PK and pharmacodynamic standpoint, drugs that exhibit concentration-dependent killing are most effective when higher dose levels are administered infrequently (33–35). For example, a 400-mg dose of CD101 achieves higher *C*_max_ values (4.0-, 3.0-, and 2.6-fold), higher AUC values in the first 48 h (2.9-, 1.6-, and 2.6-fold), and higher AUC values in the first week (1.8-, 1.4-, and 1.9-fold) than a 100-mg daily dose of micafungin (36), a 200-mg loading dose followed by a 100-mg daily dose of anidulafungin (37), and a 70-mg loading dose followed by a 50-mg daily dose of caspofungin (38), respectively. Indeed, a recent poster presented by Bader et al. (39) suggests that the dosing regimens afforded by currently available echinocandins were suboptimal. Front-loading antimicrobial drug exposure maximizes the drug effect early in the course of therapy to increase the rate and extent of pathogen killing, reduce spontaneous mutations, eliminate preexisting drug-resistant subpopulations, and improve outcomes in critically ill patients. It has been demonstrated that rapid initiation of appropriate antifungal therapy can reduce mortality in patients with candidemia (40–43). Given its PK profile (long *t*_1/2_ and low Cl) and favorable clinical safety profile, extensive therapeutic window in animal models (26), and concentration-dependent killing *in vitro* and *in vivo* (30, 32), a front-loaded dosing regimen for CD101 may maximize the drug effect, reduce drug resistance, facilitate outpatient use, and improve patient outcomes.

### Conclusions.

Safety results from two phase 1, randomized, double-blind, placebo-controlled, dose-escalation studies in healthy adult subjects indicate that single and multiple doses of CD101 IV administered at up to 400 mg once weekly for 3 consecutive weeks were safe and well tolerated. These studies also define the PK profile of single and multiple doses of CD101 in healthy subjects. CD101 exhibits a long plasma *t*_1/2_, low Cl from the body, minimal accumulation over several weeks, and negligible renal excretion and demonstrates plasma exposures that enable the use of a once-weekly dosing regimen over several weeks. Pharmacokinetic data from these studies were used to establish appropriate front-loaded dosing regimens in ongoing efficacy studies of CD101.

## MATERIALS AND METHODS

The protocols and informed consent were reviewed and approved by an appropriate Institutional Review Board before subject enrollment. The studies were designed and monitored in compliance with the ethical principles of good clinical practice and in accordance with the Declaration of Helsinki.

### Study design and treatment.

The single-ascending-dose and multiple-ascending-dose studies were phase 1, randomized, double-blind, dose-escalation, placebo-controlled, single-center safety trials of CD101 IV in healthy adults (ClinicalTrials.gov identifiers: NCT02516904 for the single-ascending-dose study; NCT02551549 for the multiple-ascending-dose study). In the single-ascending-dose study, 4 cohorts (corresponding to doses of 50, 100, 200, and 400 mg) of 8 subjects (6 active and 2 placebo) received CD101 IV or placebo infused over 60 (±5) min. In the multiple-ascending-dose study, 3 cohorts (cohort 1, 100 mg ×2 doses; cohort 2, 200 mg ×2 doses; cohort 3, 400 mg ×3 doses) of 8 subjects (6 active and 2 placebo) received CD101 IV or placebo (normal saline solution) infused over 60 (±5) min, with doses separated by 7 days.

### Inclusion criteria.

Randomized subjects were 18 to 55 years of age, in good health, and without signs or symptoms of illness. Inclusion criteria included the absence of physical examination findings that would interfere with interpretation of study results; screening ECG results without clinically significant abnormalities; clinical chemistry, hematology, and urinalysis (UA) results within reference ranges; laboratory values outside the reference range but clinically insignificant; body mass index values of 18.5 to 32.0 kg/m^2^; and negative urine screen results for drugs of abuse.

### Exclusion criteria.

Exclusion criteria included pregnant or nursing females; females of childbearing potential; positive screen results for hepatitis B virus surface antigen, hepatitis C virus, or human immunodeficiency virus antibody; current smoker; and receipt of prescription medications within 28 days or of over-the-counter medications (except for acetaminophen) within 14 days before dosing.

### Study population.

Analysis populations were intent to treat (randomized subjects), safety (subjects who received any amount of study drug), and PK analysis (subjects who received CD101 IV and had any blood or urine samples analyzed).

### Safety assessments.

All subjects were monitored for AEs throughout the study until 21 days after the last dose of study drug. Electrocardiograms, hematology and serum chemistry evaluations, and UA were performed at screening, on day −1, and at various time points during and for 14 days after the dosing period. Subjects remained in the clinical research unit for observation and safety assessments from day −1 until 7 days after the last dose of study drug. Three additional safety assessments were performed at weekly follow-up visits following the last dose of study drug. The long follow-up period ensured that any delayed safety events were captured, since CD101 has a very long *t*_1/2_ and plasma levels are expected to persist for several weeks. Safety was assessed ≥5 days after dosing by study physicians to determine whether it was safe to proceed to the next dose.

### Pharmacokinetic assessments.

Extensive PK sampling from plasma and urine was performed for all subjects following study drug administration and over the 3 weeks after the last dose of study drug. Plasma and urine samples from subjects who received CD101 IV were analyzed by a bioanalytical laboratory for CD101 concentration using a validated liquid chromatography-tandem mass spectrometry method with a lower limit of quantitation of 10 ng/ml.

### Statistical methods.

Both studies were exploratory and not powered for inferential statistical analyses.

Descriptive statistics, including the numbers and percentages for categorical variables and the numbers, means, standard deviations (SD), and medians, minimums, and maximums for continuous variables, were summarized by cohort (i.e., dose) and study drug (CD101 IV or placebo). A statistical analysis plan was prepared and finalized before database lock and analysis of data were performed. All statistical analyses were performed using SAS Version 9.3.

Safety was assessed in the safety analysis population. Safety was evaluated by presenting summaries of AEs, ECGs, clinical laboratory evaluations (hematology evaluation, chemistry panel, and UA), and vital signs. Adverse events were coded using the *Medical Dictionary for Regulatory Activities* Version 18.0. A TEAE was an AE that occurred during or after study drug administration and up through 21 (±1) days after study drug administration. The incidence of TEAEs was summarized by system organ class and preferred term, by relationship to study drug, and by severity. In addition, the incidence of serious TEAEs and the incidence of TEAEs leading to discontinuation of study drug were summarized by system organ class and preferred term. Descriptive statistics for clinical laboratory test results and vital signs, and for changes from baseline, were summarized by time point. Incidences of potentially clinically significant clinical laboratory results and vital signs were also summarized by time point. The numbers and percentages of abnormal ECGs were summarized by time point.

Pharmacokinetics were assessed in the PK analysis population and included *C*_max_, AUC, Cl, elimination rate constant (λz), and terminal *t*_1/2_. Plasma concentration-time profiles were constructed for each subject and dosing cohort. For each plasma concentration-time curve, PK parameters were determined directly from inspection of the data or calculated using noncompartmental methods with validated PK software (Phoenix WinNonlin, Version 6.3). Urine concentration data were used to determine renal excretion and drug recovery rates. The number of subjects was selected to allow sufficient evaluation of safety, tolerability, and PK and was consistent with phase 1 standards of practice (44).
